# Association between chronic pain and dementia: a systematic review and meta-analysis

**DOI:** 10.1007/s10433-024-00812-2

**Published:** 2024-05-22

**Authors:** Zhenzhi Wang, Zhen Sun, Hui Zheng

**Affiliations:** 1 The Acupuncture and Tuina School, Chengdu University of Traditional Chinese Medicine, No. 1166 Liutai Avenue, Wenjiang District, Chengdu, 611100 China; 2 State Key Laboratory of Oncogenes and Related Genes, Shanghai Cancer Institute, Renji Hospital, Shanghai Jiao Tong University School of Medicine, Shanghai, China

**Keywords:** Chronic pain, Dementia, Meta-analysis, Systematic review

## Abstract

**Purpose:**

Dementia and chronic pain (CP) are prevalent among older adults. However, no study has systematically reviewed the association between dementia and CP. Therefore, we performed this study to gather evidence about the potential relationship between the two.

**Methods:**

Two authors independently searched PubMed, Embase, and Web of Science to identify all records published up to 1 September 2022 that explored the association between CP and dementia. The methodological quality of the studies was assessed using the Newcastle Ottawa Scale (NOS). A fixed or random-effects model was used to pool the risk estimates.

**Results:**

Among the initial 3296 articles retrieved, 19 were included in the review (1 cross-sectional, and 18 cohort). The pooled result showed the risk of dementia was 1.42 times higher in CP patients (HR = 1.42, 95% CI 1.23–1.64, *P* < 0.001). dementia and CP subtypes, gender, and age did not significantly affect the results.

**Conclusion:**

Our study shows that people who suffered from CP are at an increased risk of developing dementia, regardless of gender, age, and dementia and CP subtypes.

**Supplementary Information:**

The online version contains supplementary material available at 10.1007/s10433-024-00812-2.

## Introduction

Chronic pain (CP) is defined by the International Association for the Study of Pain (IASP) as pain that lasts for more than 3 months (Turk et al. 2011; International Association for the Study of Pain 1986). It affects over 30% of people worldwide and is associated with secondary disabilities and comorbidities such as anxiety, depression, and suicide (Cohen et al. 2021; Vos et al. 2012; Dueñas et al. 2015; Fayaz et al. 2016). Arthritis and primary chronic pain syndromes are among the most common types of chronic pain conditions (Ossipov and Porreca 2006). The elderly population, particularly those over 65 years old, are particularly prone to CP, leading to significant suffering, including distress, social isolation, disability, and increased healthcare costs (Dahlhamer et al. 2018; Tsang et al. 2008; Patel and Guralnik 2013; Leveille et al. 2009; Pitcher and Korff 2018; Bernfort et al. 2015). Early identification and effective treatment of pain can greatly improve the quality of life for older adults, reducing the risk of falls, agitation, depression, and anxiety (Hellstrom et al. 2004; Smalbrugge et al. 2007; Frederick 2016). Therefore, the relief of pain holds great importance in both clinical practice and scientific research.

There is growing evidence indicating that the health consequences of pain are associated with the onset and progression of dementia (Whitlock et al. 2017; Belin and Gatt 2006; Wang et al. 2022; Wang and Liu 2021; Khalid et al. 2022, 2020; Innes and Sambamoorthi 2020; Whitlock et al. 2017 Aug 1b; Rouch et al. 2022; Tzeng et al. 2018; Cheng et al. 2022; Kao et al. 2021; Hurh et al. 2022; Morton et al. 2019; Hagen et al. 2014; Islamoska et al. 2020; George et al. 2020; Lee et al. 2019; Kostov et al. 2019; Chen et al. 2018; Huang et al. 2015), which is a critical issue. Neurodegenerative diseases, such as Alzheimer’s disease and related dementias, are increasingly prevalent worldwide due to the rapid aging of the global population. This poses a significant and urgent public health crisis (Abubakar et al. 2015; Feigin et al. 2017; Alzheimer’s Association 2021; Sanders and Morano 2008), leading to disability, institutionalization, and premature mortality. Dementia imposes a substantial burden on individuals, society, and the economy. By screening for dementia and implementing preventative and therapeutic interventions, utilizing pain as a risk factor, we aim to alleviate the burden of disability, reduce rates of institutionalization, and extend life expectancy. To explore the relationship between chronic pain and dementia, we conducted a systematic review and meta-analysis. 

## Methods and materials

This meta-analysis was reported following the Preferred Reporting Items for Systematic Reviews and Meta-analyses (PRISMA 2020) statement (Page et al. 2021).

### Search strategy

A systematic search was conducted on PubMed, Embase, and Web of Science to identify studies related to chronic pain and dementia (Online Appendix 1). The following search terms were used: “Chronic pain,” “Pains, Chronic,” “Widespread or Chronic Pains,” “Intractable Pain,” “Refractory Pains,” “Pain, Refractory,” “Dementia,” “Alzheimer’s Disease,” “Alzheimer’s Type Dementia,” “ATD,” and “Alzheimer Type Dementia, Early Onset.” The literature search covered articles up until September 1, 2022. Two researchers independently screened the literature, and in cases where consensus could not be reached, a third researcher reviewed the full text and resolved any differences. There were no language restrictions or filters applied to the search. Additionally, we checked the bibliographies of relevant reviews, articles, and books for further references.

### Inclusion and exclusion criteria

The studies included in the meta-analysis had to meet the following criteria: (a) being observational case–control, cohort, or cross-sectional studies; (b) focusing on chronic pain or specific subtypes of chronic pain as the primary exposure factor associated with the risk of dementia; (c) reporting effect estimates with corresponding 95% confidence intervals (CIs), or providing sufficient data to calculate crude risk ratios (HRs) for the association between chronic pain and dementia. Studies were excluded if they met any of the following criteria: (a) being published only as letters or abstracts; (b) not reporting quantitative outcome data; (c) not published in English; (d) being case reports; and (e)studies were excluded if they lacked access to the complete set of literature or if the full text was not obtainable.

### Study selection and data extract

Two authors (WZZ and SZ) will independently extract data from the included studies. Before starting the review process, a pre-made Excel datasheet will be used to collect the following information: general details (title, author, country of study, year of publication), study characteristics (study design, inclusion and exclusion criteria), study population (age, sex, sample size, number of analyses, type of chronic pain, dementia subtype), exposure characteristics (type of chronic pain, duration, duration of follow-up), and outcomes (primary and secondary outcomes, time points, methods of outcome evaluation). If necessary, the authors of the original trials will be contacted for additional information and clarification of the data. Any discrepancies or disagreements will be resolved through discussion and consensus.

### Statistical analysis

The data analysis for this study was conducted using Stata 17.0. Hazard ratios (HRs) and corresponding 95% confidence intervals (CIs) were used to assess the predictive effect of chronic pain on the risk of dementia. Heterogeneity between studies was assessed using Cochran’s Q-test and Higgins’ I^2^ statistics. If I^2^ and *p*-value for heterogeneity were greater than 0.05, fixed effects models were used to pool the results. In cases where significant heterogeneity existed, random-effects models were employed (Higgins et al. 2003). Univariate meta-regression analysis was conducted to identify potential sources of heterogeneity. Subgroup analyses were performed based on dementia subtype, chronic pain subtype, country, age (mean/median year < 65 or ≥ 65), severity, gender (male, female), follow-up time, education level (low, medium, high), and study design.

If there was insufficient validity data provided in a study for meta-analysis, a narrative approach was used to describe the relevant findings. Sensitivity analysis was conducted by excluding one study at a time to evaluate the stability of the results. Additionally, multiple studies were excluded simultaneously in sensitivity analysis to determine the robustness of the findings. Publication bias was assessed by examining funnel plot asymmetry and conducting Egger’s test and Begg’s test (significance threshold: *p* < 0.05). If both the p-values and funnel plots showed symmetry, it indicated no publication bias (Egger et al. 1997).

### Study quality assessment

The quality of the included cohort studies was evaluated using the Newcastle–Ottawa Scale (NOS), which assesses three components: selection, comparability, and outcome. A NOS score of 7 indicates high quality, while a lower score suggests a higher risk of bias (Stang 2010). For the included cross-sectional studies, the quality assessment was conducted using the Agency for Healthcare Research and Quality (AHRQ) criteria. One author assessed the quality of individual studies, and another author independently checked the assessments. Any disagreements were resolved through consensus.

## Results

### Study characteristic

The included studies in this meta-analysis were conducted across eight countries: the United States (Wang et al. 2022; Wang and Liu 2021; Khalid et al. 2022, 2020; Innes and Sambamoorthi 2020; Whitlock et al. 2017; George et al. 2020), China (Tzeng et al. 2018; Cheng et al. 2022; Kao et al. 2021; Chen et al. 2018; Huang et al. 2015), France (Rouch et al. 2022), the United Kingdom (Kostov et al. 2019), Korea (Kao et al. 2021; Lee et al. 2019), Norway (Hagen et al. 2014), Canada (Morton et al. 2019), and Denmark (Islamoska et al. 2020). All of these studies were population-based and involved a total of 842,806 subjects, including 132,445 patients with chronic pain. Among the included studies, one was cross-sectional (Wang et al. 2022) and two were case–control designs (Lee et al. 2019; Chen et al. 2018), while the remaining 16 studies were cohort studies. Eighteen studies examined the risk of developing dementia after a diagnosis of chronic pain (Wang and Liu 2021; Khalid et al. 2022, 2020; Innes and Sambamoorthi 2020; Whitlock et al. 2017; Rouch et al. 2022; Tzeng et al. 2018; Cheng et al. 2022; Kao et al. 2021; Hurh et al. 2022; Morton et al. 2019; Hagen et al. 2014; Islamoska et al. 2020; George et al. 2020; Lee et al. 2019; Kostov et al. 2019; Chen et al. 2018; Huang et al. 2015), and one study explored the comorbidity of dementia in patients with chronic pain (Wang et al. 2022).

Diagnosis of chronic pain and dementia in the medical records databases relied on diagnostic codes such as the International Classification of Diseases. The gender distribution was generally balanced across the included studies, but the median/mean age varied among the studies, mostly being over 50 years, except for two studies where age information was not reported (Islamoska et al. 2020; Huang et al. 2015), and one study that included individuals aged > 20 years (Hagen et al. 2014). Fifteen cohort studies had follow-up periods ranging from 2 to 16 years, while one study did not provide follow-up duration (Wang et al. 2022; Lee et al. 2019; Chen et al. 2018). Age, gender, and comorbidities were matched and/or adjusted for in all studies. Table 1 provides detailed information for each study.

**Table 1:** General study characteristics of the included articles

Study, year	Design	Country	Sources of participants	No. (expose/ unexposed)	Occupation	Mean/Median age years	Chronic Pain description	Reference category definition	Exposure duration (mean, years)	Exposure intensity (No, Mild, Moderate Severe)	Follow-up (CP/control) (years)	Diagnosis of Dementia/AD	Adjustment Variables	Subgroup analysis
Wang, 2022	cross-sectional	America	Health and Retirement Survey	16,380 (1714/14666)	No reported	67	Questions about if they were often troubled by pain, and evaluate the intensity of their pain on a 3-point scale (No / Mild / Moderate / Severe)	No pain	NA	No, Mild, Moderate Severe	NA	Assessment of the respondent’s memory	Age gender, marital status, race/ethnicity, highest educational degree, employment status, number of children living in the same household, insurance coverage (Medicare, Medicaid, and Private), self-rated health status, ADL limitations, IADL limitation, depressive symptoms, specific chronic conditions (diabetes, osteoarthritis, stroke, cancer, lung disease, heart disease, emotional)	Pain, Reported Pain Severity (No / Mild / Moderate / Severe)
Wang, 2021	Cohort	America	Framingham Heart Study (FHS) population	2464 (347/2117)	No reported	≥ 65	All participants were asked if the pain, aching or stiffness in any of their joints occurred on most days	No WSP	NA	NA	10	Dementia: the Diagnostic and Statistical Manual of Mental Disorders, Fourth Edition; AD: the National Institute of Neurological and Communicative Disorders	Sociodemographic plus health status and health behaviors): adjusted for age, sex, SBP, treatment of hypertension, TC, prevalent diabetes mellitus, current smoking, alcohol consumption, analgesic history, depression scale, employment status, dietary fiber intake, personal income, marital status, and BMI, and all-cause dementia and AD dementia were additionally adjusted for MMSE at baseline and education level	Subtypes of dementia (Vascular dementia, AD, Other dementia) WSP
Khalid, 2022	Cohort	America	ADRD-free West Virginia (WV) Medicare fee-for-service beneficiaries	161,573 (65,891/ 95,682)	No reported	≥ 65	Two outpatient claims (90 days apart) or one inpatient claim using ICD-9-CM codes as recommended by CMS	No CNCP	NA	NA	3	At least one FFS claims with any of the following International Classification of Diseases, Ninth Edition, clinical modification (ICD-9-CM) diagnostic codes: 290.0–290.3, 331.0–331.2, 331.7, and 331.8 (Lin et al., 2010) or ICD-10-CM codes: G30.xx and F02.xx”	Age group, sex (female/male), race/ethnicity, smoking, status (current smoker/non-smoker) and obesity (yes/no), history of stroke or traumatic brain injury (TBI); and chronic health conditions, including hypertension, diabetes, heart disease, respiratory illness, and cancer, as well as specific auto-immune conditions associated with chronic pain, including rheumatoid arthritis (RA), and systemic lupus erythematosus (lupus). Analgesic use, defined as baseline use (yes/ no) of non-steroidal anti-inflammatory medications (NSAIDS) and opioid analgesics, was ascertained using records of reimbursed prescription claims during the baseline year	Number of CNCP
Khalid, 2020	Cohort	America	Medicare Current Beneficiary Survey (MCBS)	16,934 (6369/ 10,565)	No reported	≥ 65	CNCP included five common CNCP (back or neck pain, headache, joint pain, neuropathic pain, and osteoarthritis)	No CNCP	NA	NA	2	ICD-9-CM 332	Sex, race/ethnicity, marital status educational level, family income, measured as the percentage of the federal poverty line (FPL), health insurance status, private insurance, rurality, and lifestyle factors, smoking, status, and body mass index (BMI)	Number of CNCPs
Innes, 2020	Cohort	America	Medicare Current Beneficiary Survey (MCBS)	16,934(4545/ 12,389)	No reported	≥ 65	The presence of two or more outpatient claims, at least 90 days apart, for the specific condition or anyone inpatient or skilled nursing facility claim	No OA	NA	NA	4–10	1) one or more FFS claims with any of the following International Classification of Diseases, Ninth Edition, clinical modification (ICD-9-CM) diagnostic codes: 290.0–290.3, 331.0–331.2, 331.7, and 331.8; 2) an affirmative response to the self-reported Health Status question “Has a doctor ever told you that you had Alzheimer’s?’	Age group, sex, race/ethnicity, education, marital status, family income, measured as the percentage of the federal poverty line (FPL), health insurance status, private insurance, rurality, smoking status (current smoker, past smoker, never smoked) and body mass index (BMI)	OA
Whitlock, 2017	Cohort	America	Health and Retirement Study (HRS)	10,065(1120/8945)	No reported	67–78	Questions about if they were often troubled by pain, and evaluate the intensity of their pain on a 3-point scale (mild, moderate, severe)	No Pain	NA	NA	10	Dementia probability incorporates direct and proxy cognitive test responses to estimate the chance that an individual would meet the Diagnostic and Statistical Manual of Mental Disorders (Third Edition Revised) or Diagnostic and Statistical Manual of Mental Disorders (Fourth Edition) diagnostic criteria for dementia	Age, sex, race/ethnicity, education tobacco use, medical comorbidities, total household financial assets, marital status, current alcohol use, depressive symptoms	Persistent Pain
Rouch, 2021	Cohort	French	A subsample of the PAQUID population	593 (79/514)	No reported	≥ 65	Questions about if they experienced any pain anywhere, and evaluate the location, severity, frequency, and frequency of their pain on a 3-point scale (mild, moderate, severe). The participants with at least moderate or intense daily pain for more than 6 months were considered as having chronic pain (CP +)	Participants who reported no pain or mild pain, occurring infrequently, or moderate or severe daily pain for less than a month (CP −)	More than 6 months	Mild, moderate, severe	11.3	the neuropsychologist selected the participants who were suspected of having dementia by completing a criteria checklist for dementia using the Diagnostic and Statistical Manual of Mental Disorders, Third Edition, Revised (DSM‐III R) checklist. The participants who met dementia criteria, or had at least a three‐point decline in the mini‐mental status examination score at their previous visit, were re‐evaluated at home by a senior neurologist or geriatrician to confirm or exclude the diagnosis	Sex, education, depressive symptomatology, antidepressant use, number of comorbidities, and number of visits	Chronic pain
Tzeng, 2017	Cohort	China	Taiwan’s National Health Insurance Research Database (NHIRD)	166,448 (41,612/124836)	No reported	≥ 50	Al diagnosis of fibromyalgia is made by rheumatologists, internists, neurologists, or pain medicine specialists	Without fibromyalgia, matched for exact age, gender, and index year	NA	NA	10	All diagnoses of dementia in Taiwan are made by board-certified psychiatrists or neurologists	Gender, age, monthly income, urbanization level, geographic region of residence, and comorbidities	Fibromyalgia, Comorbidity, Urbanization level, Insured premium, Medication
Cheng, 2022	Cohort	China	Taiwan National Health Insurance Research Database (NHIRD)	3810 (762/3048)	No reported	≥ 50	a Record of an ICD-9-CM code 350.1 assigned by a neurologist in two or more consecutive ambulatory visits or in one or more hospital inpatient stays	no TN	NA	NA	15	Using the International Classification of Diseases, Ninth Revision, Clinical Modification (ICD-9-CM) diagnosis classifications of 290, 294.1, 294.2, or 331 with at least one hospitalization or 3 outpatient clinic visits	Age, sex, Charlson comorbidity index score, relevant comorbidities, and medications	TN, sex, age
Kao, 2021	Cohort	China	Taiwan’s National Health Insurance (NHI)	55,584 (27,792/27792)	No reported	≥ 50	Patients who used analgesics for at least 3 months	No Migraine	NA	NA	5	AD (ICD-10 codes G30 or F00), VAD (ICD-10 code F01), other specified dementias (ICD-10 codes F02, G31.00, G31.82), and unspecified dementia (ICD-10 code F03)	Comorbidities	Migraine
Hurh, 2022	Cohort	Korea	Korea National Health Insurance Service Health Screening Cohort (NHIS-HEALS)	88,390 (44,195/44195)	No reported	55.3 ± 9.4	ICD-10: G43	No Migraine	NA	NA	16	AD (ICD-10 codes G30 or F00), VAD (ICD-10 code F01), other specified dementias (ICD-10 codes F02, G31.00, G31.82), and unspecified dementia (ICD-10 code F03)	NA	Migraine, Subtypes of dementia (All- cause dementia, AD, Dementia)
Morton, 2019	Cohort	Canada	The Canadian Study of Health and Aging (CSHA)	1355 (628/727)	No reported	≥ 65	NA	No Migraine	NA	NA	5	all‐cause dementia: DSM‐IV criteria, AD: NINCDS‐ADRDA criteria, VAD: NINDS‐AIREN	age, education	Migraine, age
Hagen, 2013	Cohort	Norway	Nord-Trøndelag Health Study (HUNT 2)	51,895 (21,871/29988)	No reported	≥ 20	Questions about if they had a current headache disorder and frequency of headache (1–14 days or ≥ 15 days)	Headache free	≥ 9 years, 10–12 years, or ≥ 13	NA	15	ICD-10 criteria: dementia, ICD-10 criteria: AD and VAD	Age, gender, other potential confounding factors identified previously, current use of antihypertensive medication, physical activity, BMI, total HADS score, self-reported cardiovascular diseases, abstention from alcohol, total cholesterol, triglycerides (continuous variable), nonfasting glucose, daily use of any medication, and self-reported stroke	Migraine
Islamoska, 2020	Cohort	Denmark	national register-based data on all inhabitants in Denmark of all ethnic origins born	62,578 (10,857/51721)	No reported	NA	Hemicrania ophthalmoplegia (ICD-8:346.00), ICD-8: 346.08, ICD-8: 346.09; migraine (ICD-10: G43), MO (ICD-10: G43.0), MA (ICD-10: G43.1), status migrainosus (ICD-10: G43.2), complicated migraine (ICD-10: G43.3), other migraine (ICD- 10: G43.8), or unspecified migraine (ICD-10: G43.9)	No Migraine	NA	NA	Median:6.9	ICD-8 and ICD-10	Birth date, sex, country of origin, marital status, highest educational level	Migraine, Follow-up period (year), Educational level, sex
George, 2020	Cohort	America	Atherosclerosis Risk in Communities (ARIC) study	12,495 (2640/9855)	No reported	51–70	1) headache lasting 4 or more hours; (2) headache with throbbing, pounding, or pulsating1) headache lasting 4 or more hours; (2) headache with throbbing, pounding, or pulsating pain or that was unilateral; (3) symptoms of nausea, vomiting, or sensitivity to light or sound; and (4) one or more years with a history of headaches	Participants who denied having a headache lasting 4 or more hours were classified as having no headache	Lasting 4 or more hours	NA	Median:21	ICD codes	Age, sex, race center, APOE ε4, income, and education from visit 1, BMI, smoking status, hypertension, diabetes, CHD, drinking status, HDL, and total cholesterol	Migraine, sex
Lee, 2018	Case–control	Korea	Korean National Health Insurance Service (NHIS)-National Sample Cohort	57,190 (11,438/45752)	No reported	≥ 60	ICD-10: G43	No Dementia	58.6 months	NA	NA	Diagnoses of Alzheimer's disease (G30) , dementia in Alzheimer’s disease (F00)	Ischemic heart disease, and cerebral stroke	Migraine, age, and sex
Kostev, 2019	Cohort	United Kingdom	Dis-74 ease Analyzer database (IQVIA)	7454 (3727/3727)	No reported	67.7	ICD-10: G43	No Migraine	NA	NA	10	ICD-10: F01, F03, G30	NA	Migraine, Subtypes of dementia (All-cause dementia, Vascular dementia, AD, Unspecified dementia
Chen, 2018	case–control	China	Taiwan’s National Health Insurance Program (NHI pro- gram)	71,260 (10,180/61080)	No reported	≥ 40	ICD-9-CM:715	No OA	NA	NA	NA	ICD-9-CM: 290.0–290.4, 294.1,331.0	NA	OA, sex, age
Huang, 2015	Cohort	China	Taiwan Longitudinal Health Insurance Database 2005 (LHID2005)	105,447 (35,149/70298)	No reported	NA	ICD-9-CM: 715	No OA	NA	NA	4	CD-9-CM:290, 294, and 331	Age and sex, DM, hyperlipidemia, hypertension, coronary heart dis- ease, COPD, stroke, and Parkinson’s disease in hospitals or clinics	OA

*NA* Not applicable, *OA* Osteoarthritis, *TN* Trigeminal neuralgia, *WSP* Widespread pain, *CNCP* Chronic non-cancerous pain

Table 2 shows that all cohort studies are of high quality (Scores of 8 to 11 are considered high quality). Despite the absence of follow-up data, this is because the cross-sectional design does not require follow-up data (Wang et al. 2022).

**Table 2:** Quality assessment of included studies

Study (cohort)	Representativeness of exposed cohort	Selection of non-exposed cohort	Ascertainment of exposure	Outcome not present before study	Comparability	Assessment of outcome	Follow-up long enough	Adequacy of follow up	Quality score
Wang, 2021	*	*	*	*	**	*	*	*	9
Khalid, 2022	*	*	*	*	**	*		*	8
Khalid, 2020	*	*	*	*	**	*		*	8
Innes, 2020	*	*	*	*	**	*	*	*	9
Whitlock, 2017	*	*	*	*	**	*	*	*	9
Rouch, 2021	*	*	*	*	**	*	*	*	9
Tzeng, 2017	*	*	*	*	**	*	*	*	9
Cheng, 2022	*	*	*	*	**	*	*	*	9
Kao, 2021	*	*	*	*	**	*	*	*	9
Hurh, 2022	*	*	*	*	**	*	*	*	9
Morton, 2019	*	*	*	*	**	*	*	*	9
Hagen, 2013	*	*	*	*	**	*	*	*	9
Islamoska, 2020	*	*	*	*	**	*	*	*	9
George, 2020	*	*	*	*	**	*	*	*	9
Kostev, 2019	*	*	*	*	**	*	*	*	9
Huang, 2015	*	*	*	*	**	*		*	8

Study (case–control)	Case definition	Representativeness of the cases	Selection of Controls	Definition of Controls	Comparability	Ascertainment of exposure	Same method	Non-Response rate	Quality score
Lee et al. 2019	*	*	*	*	**	*	*	*	9
Chen, 2018	*	*	*	*	**	*	*	*	9

Follow-up long enough: *Median/mean follow-up of more than 5 years or maximum follow-up of more than 10 years was considered enough

Adequacy of follow-up: *A follow-up rate of > 80% and a descriptive analysis of those who were missing were considered adequate

## Results

**Fig. 1:**
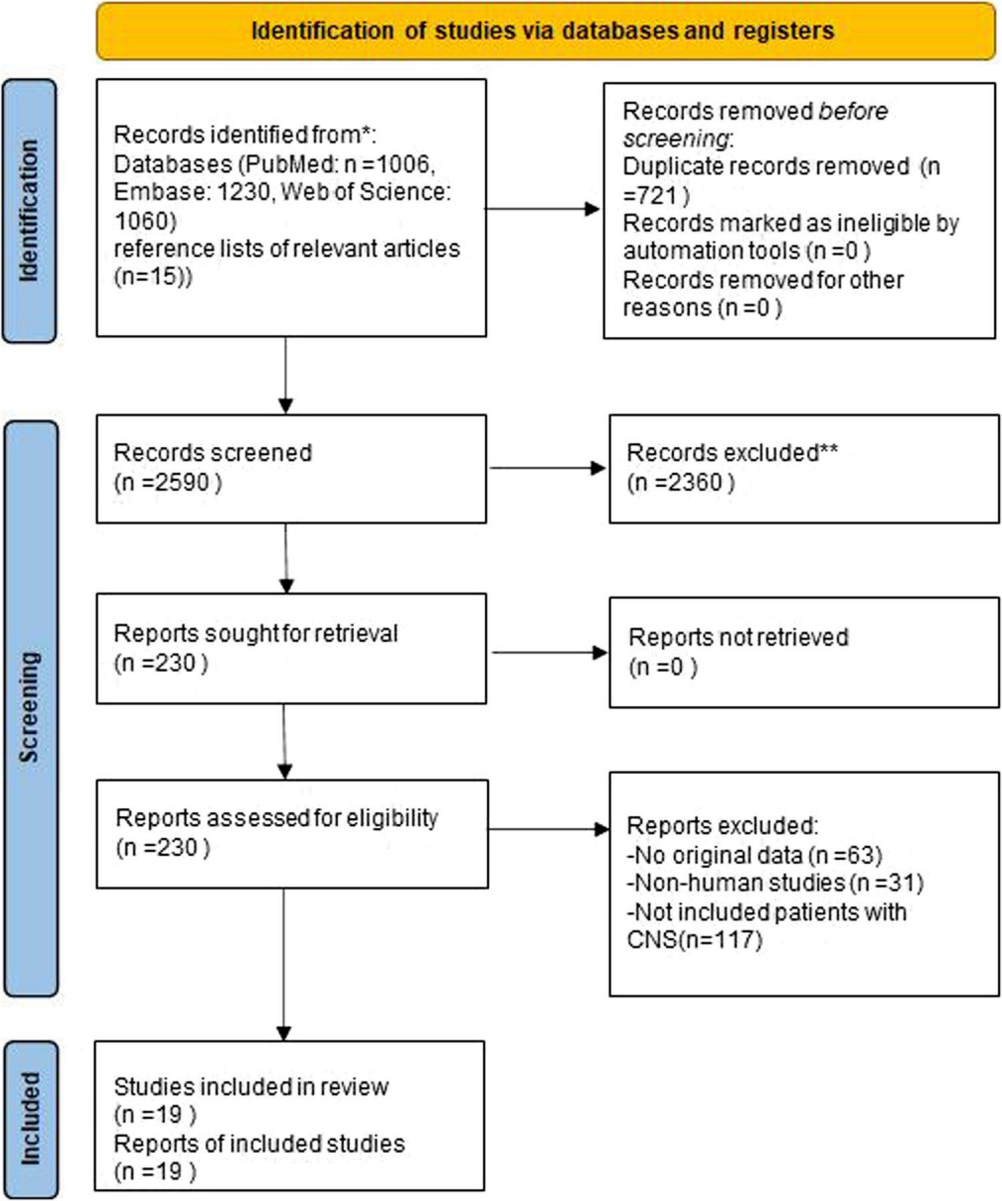
Flow diagram of the study selection process

A total of 3311 potentially relevant papers were identified through the pre-developed search strategies. After removing 3025 duplicate records, the titles and abstracts of 3081 remaining records were screened for relevance. Following this screening process, 19 studies were included in this systematic review after a full-text reading of 230 papers. The study selection process and the reasons for exclusion after full-text reading are presented in Fig. 1.

### The association between chronic pain and risk of dementia

**Fig. 2:**
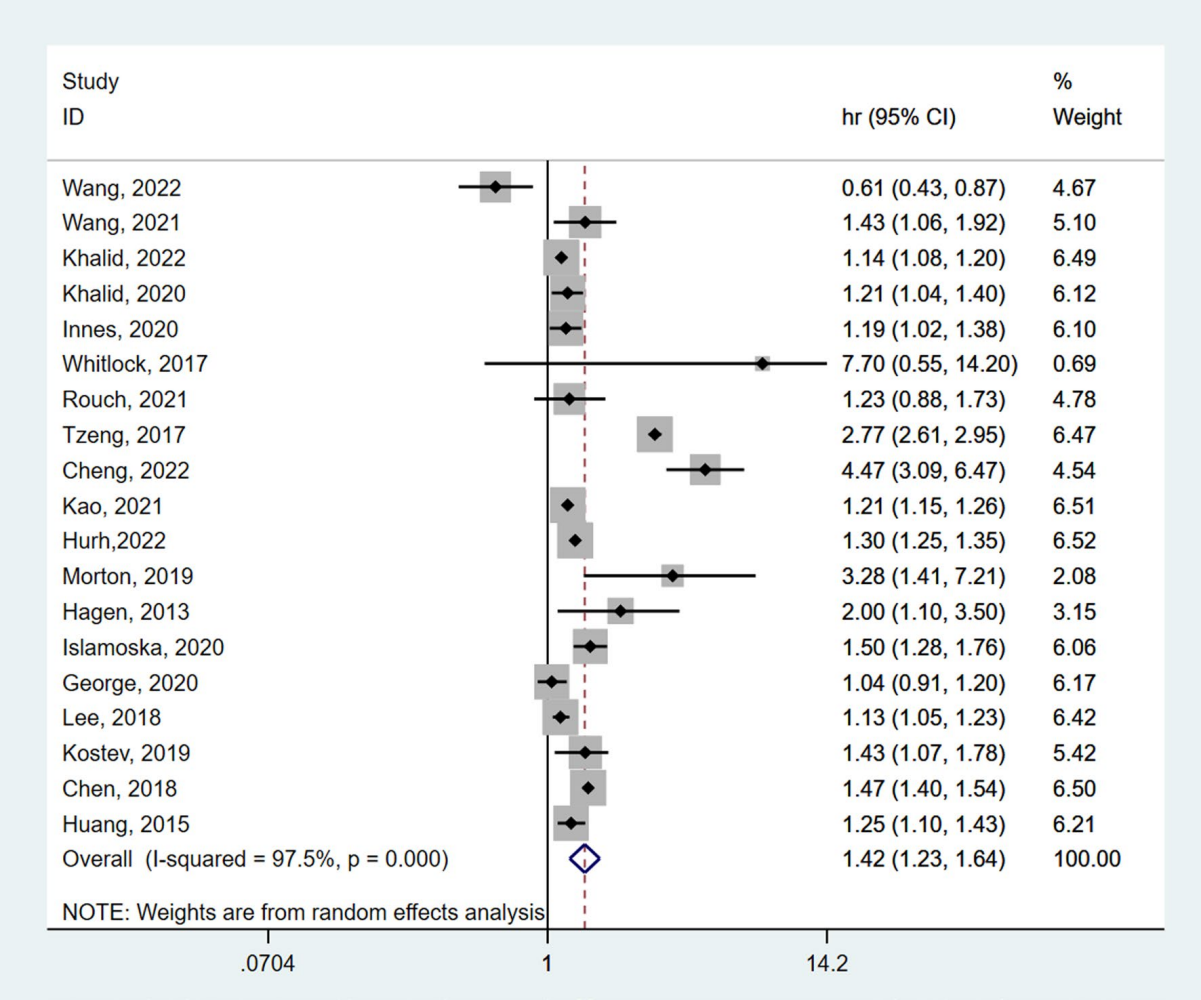
Forest plot of the relationship between CP and dementia risk

A total of 19 studies involving 908,849 participants were included in the overall meta-analysis, heterogeneity testing indicated substantial heterogeneity among studies (I^2^ = 97.5%, *P* < 0.001), so the random-effects model was applied. The pooled results showed a 42% increased risk of dementia in CP participants. (HR = 1.42, 95% CI 1.23–1.64, *P* < 0.001) **(**Fig. 2**)**.

### Subgroup analyses

Stratified analyses were conducted to examine the potential effects of various factors on the association between dementia and chronic pain. These factors included subtypes of dementia, subtypes of chronic pain, country of study, study design, follow-up time, age, and gender.

Subgroup analyses showed that Trigeminal Neuralgia (HR = 4.47, 95% CI 3.09–6.47, *P* < 0.001), Fibromyalgia (HR = 2.77, 95% CI 2.61–2.95, *P* < 0.001), Widespread pain (HR = 1.43, 95% CI 1.06–1.92, *P* < 0.001), Osteoarthritis (HR = 1.31, 95% CI 1.14–1.52, *P* < 0.001), Migraine (HR = 1.26, 95% CI 1.16–1.36, *P* < 0.001), and chronic non-cancerous pain (CNCP) (HR = 1.15, 95% CI 1.09–1.21, *P* < 0.001) were significantly associated with subsequent higher dementia risk. Furthermore, Wang et al., Hurh et al., Kostev et al. explored the association between CP and the risk of different dementia subtypes. All dementia subtypes were associated with an increased risk of CP, ranging from 36 to 44%.

Only Kao et al. (2021) assessed the association between the duration of follow-up on CP and subsequent dementia risk. Their results showed that the association between CP and dementia risk was comparable between long-term follow-up (> 5 years) and follow-up periods of less than 5 years. In terms of impacts of age, the risk of dementia seems to be similar for people over 65 years of age and those under 65 years of age (HR = 1.53, 95% CI 1.21–1.93, *P* < 0.001 vs. HR = 1.33, 95% CI 1.16–1.52, *P* < 0.001, respectively). The association between CP and dementia risk was similar between the female and male subgroups (HR = 1.49, 95%CI 1.23–1.79, *P* = 0.000 vs. HR = 1.36, 95%CI 1.10–1.69, *P* = 0.005, respectively). The educational level did not affect dementia risk **(**Table 3**)**. Notably, In the country subgroup analysis, the pooled results from the studies in America (HR = 1.12, 95% CI 0.99–1.27, *P* = 0.704) and French (HR = 1.23, 95% CI 0.88–1.73, *P* = 0.230) showed no significant correlation between CP and dementia. In other countries, CP was consistently associated with higher risks of dementia. In subgroups of study type, the association between CP and dementia risk was similar in cohort studies and case–control studies but was only statistically significant in the former. Interestingly, there was no association in the cross-sectional studies.

**Table 3:** Subgroup analysis of the association between CP and the risk of dementia

Subgroup	No. of studies	I^2^	P _heterogeneity_	HR	95% CI	Pooled model	P _overall effect_
*Subtypes of dementia*							
All-cause dementia	2	0.0	1.000	1.43	1.18–1.73	Fixed-effects model	0.000
Alzheimer’s disease	3	54.6	0.111	1.44	1.17–1.77	Random-effects model	0.001
Vascular dementia	2	0.0	0.704	1.36	1.20–1.54	Fixed-effects model	0.000
*Subtypes of chronic pain*							
Widespread pain	1	NA	NA	1.43	1.06–1.92	NA	0.000
Osteoarthritis	3	81.7	0.004	1.31	1.14–1.52	Random-effects model	0.000
Trigeminal Neuralgia	1	NA	NA	4.47	3.09–6.47	NA	0.000
Migraine	8	78.0	0.000	1.26	1.16–1.36	Random-effects model	0.000
Fibromyalgia	1	NA	NA	2.77	2.61–2.95	NA	0.000
CNCP	2	0.0	0.459	1.15	1.09–1.21	Fixed-effects model	0.000
Persistent pain	1	NA	NA	7.7	0.55–14.2	NA	0.000
*Gender*							
Female	5	92.1	0.000	1.49	1.23–1.79	Random-effects model	0.000
Male	5	90.5	0.000	1.36	1.10–1.69	Random-effects model	0.005
*Age*							
< 65	3	85.8	0.001	1.55	1.18–2.03	Random-effects model	0.000
> 65	11	92.0	0.000	1.33	1.16–1.52	Random-effects model	0.000
*Follow time, years*							
≤ 1	1	NA	NA	1.57	1.32–1.87	NA	0.000
≤ 2	1	NA	NA	1.46	1.29–1.65	NA	0.000
≤ 3	1	NA	NA	1.42	1.29–1.56	NA	0.000
≤ 4	1	NA	NA	1.33	1.23–1.45	NA	0.000
≤ 5	1	NA	NA	1.32	1.22–1.42	NA	0.000
> 5	1	NA	NA	1.19	1.12–1.26	NA	0.000
*Educational level*							
Low	1	NA	NA	1.47	1.16–1.87	NA	0.000
Medium	1	NA	NA	1.45	1.11–1.89	NA	0.000
High	1	NA	NA	1.64	1.16–2.23	NA	0.000
*Country*							
America	7	73.0	0.001	1.12	0.99–1.27	Random-effects model	0.704
French	1	NA	NA	1.23	0.88–1.73	NA	0.230
China	5	99.2	0.000	1.88	1.33–2.67	Random-effects model	0.000
Korea	2	89.7	0.002	1.22	1.06–1.40	Random-effects model	0.005
Canada	1	NA	NA	3.28	1.41–7.21	NA	0.004
Norway	1	NA	NA	2.00	1.10–3.50	NA	0.019
Denmark	1	NA	NA	1.50	1.28–1.76	NA	0.000
United Kingdom	1	NA	NA	1.43	1.11–1.84	NA	0.006
*Design*							
Cohort	16	97.7	0.000	1.53	1.28–1.82	Random-effects model	0.000
Case–control	2	96.8	0.000	1.29	1.00–1.67	Random-effects model	0.052
Cross-sectional	1	NA	NA	0.61	0.43–0.87	NA	0.000

*CNCP* Chronic non-cancerous pain, *HR* Hazard ratio, *NA* Not applicable

### Sensitivity analysis and evaluation for publication *bias*

**Fig. 3:**
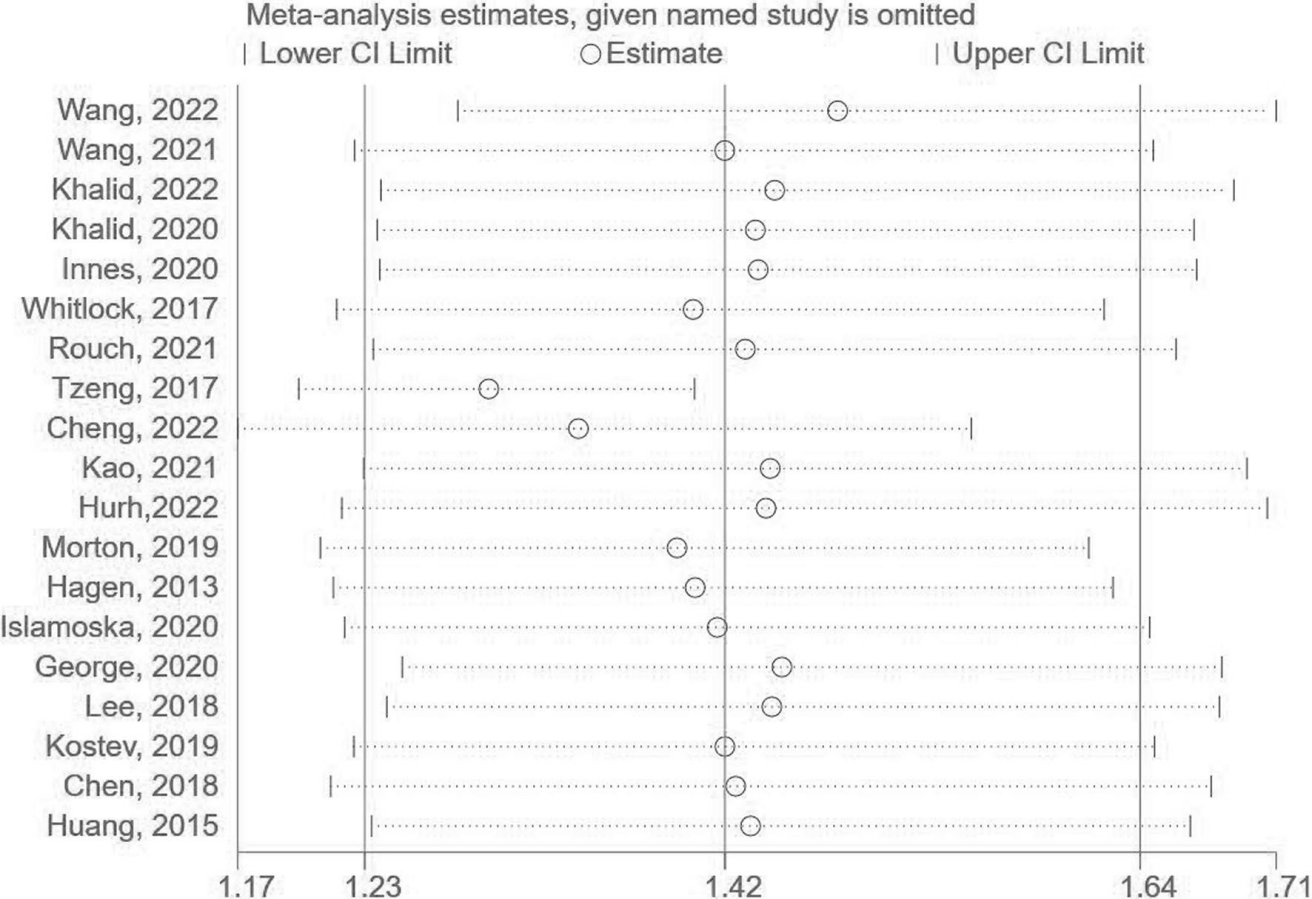
sensitivity analysis of the relationship between CP and dementia risk

**Fig. 4:**
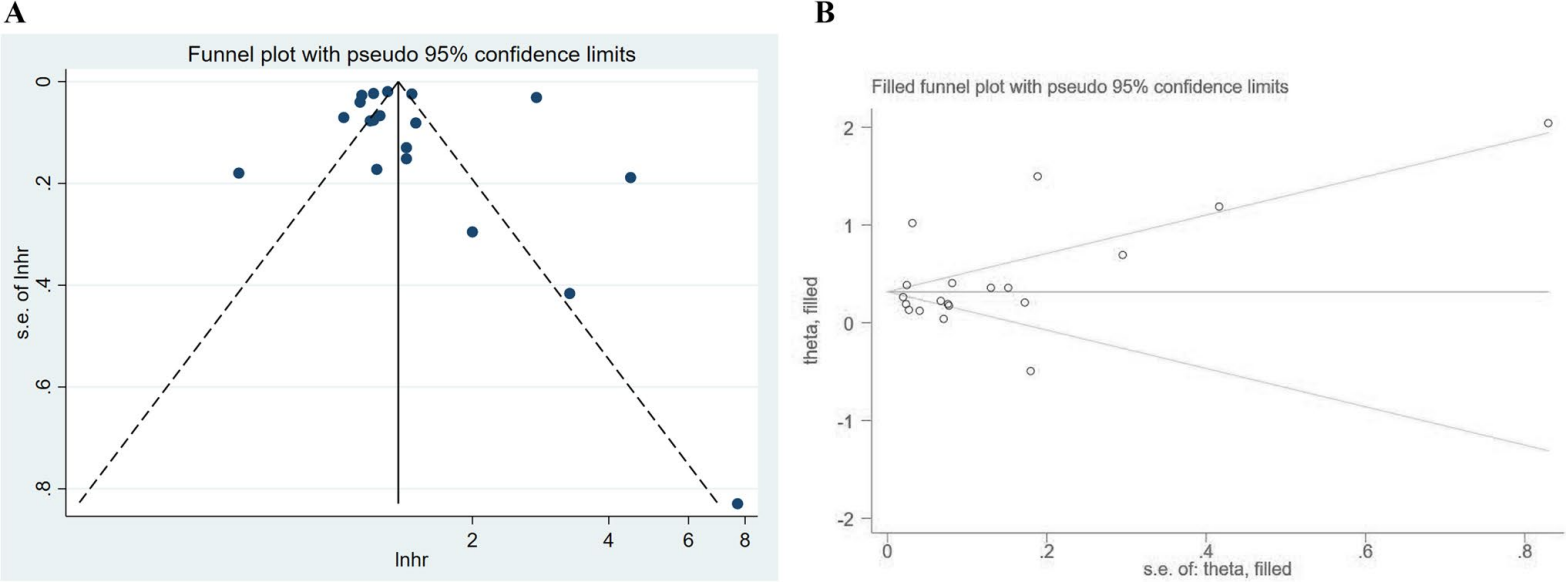
**A** Publication bias assessment; **B** Observation hypothesis study through the trim-and-fill method

A sensitivity analysis was conducted by excluding one study at a time to assess the stability of the pooled results. The analysis revealed that the effect sizes remained stable, indicating the robustness of the findings **(**Fig. 3**)**. The p-values for Begg’s and Egger’s tests were 0.036 and 0.805, respectively. The funnel plots displayed slight asymmetry, suggesting a potential publication bias **(**Fig. 4A**)**. However, no missing hypothesis studies were identified in the trim-and-fill method **(**Fig. 4B**)**, indicating that the current pooled result is relatively robust. Furthermore, due to the high heterogeneity observed, we examined the robustness of each meta-analysis by comparing the results of the random effects model with those of the fixed effects model. The pooled results of the fixed effects model showed consistent findings across all groups, except for the American group, where the results changed from insignificant to significant **(**Table 4**)**.

**Table 4:** Comparison of the results of random-effects vs. fixed-effects models

Analysis	HR (95% CI) Random-effects model	HR (95% CI) Fixed-effects model
All-cause dementia	1.43 (1.18–1.73)	1.43 (1.18–1.73)
Alzheimer’s disease	1.44 (1.17–1.77)	1.30 (1.24–1.36)
Vascular dementia	1.36 (1.20–1.54)	1.36 (1.20–1.54)
Widespread pain	1.43 (1.06–1.92)	1.43 (1.06–1.92)
Osteoarthritis	1.31 (1.14–1.52)	1.42 (1.36–1.48)
Trigeminal Neuralgia	4.47 (3.09–6.47)	4.47 (3.09–6.47)
Migraine	1.26 (1.16–1.36)	1.25 (1.21–1.26)
Fibromyalgia	2.77 (2.61–2.95)	2.77 (2.61–2.95)
CNCP	1.15 (1.09–1.21)	1.15 (1.09–1.21)
Persistent pain	7.7 (0.55–14.2)	7.7 (0.55–14.2)
Female	1.49 (1.23–1.79)	1.29 (1.24–1.79)
Male	1.36 (1.10–1.69)	1.34 (1.28–1.41)
< 65 years	1.55 (1.18–2.03)	1.40 (1.29–1.51)
> 65 years	1.33 (1.16–1.52)	1.33 (1.16–1.52)
Follow time ≤ 1 year	1.57 (1.32–1.87)	1.57 (1.32–1.87)
Follow time ≤ 2 years	1.46 (1.29–1.65)	1.46 (1.29–1.65)
Follow time ≤ 3 years	1.42 (1.29–1.56)	1.42 (1.29–1.56)
Follow time ≤ 4 years	1.33 (1.23–1.45)	1.33 (1.23–1.45)
Follow time ≤ 5 years	1.32 (1.22–1.42)	1.32 (1.22–1.42)
Follow time > 5 years	1.19 (1.12–1.26)	1.19 (1.12–1.26)
Educational low level	1.47 (1.16–1.87)	1.47 (1.16–1.87)
Educational medium level	1.45 (1.11–1.89)	1.45 (1.11–1.89)
Educational high level	1.64 (1.16–2.23)	1.64 (1.16–2.23)
America	1.12 (0.99–1.27)	1.14 (1.09–1.19)
French	1.23 (0.88–1.73)	1.23 (0.88–1.73)
China	1.88 (1.33–2.67)	1.56 (1.52–1.60)
Korea	1.22 (1.06–1.40)	1.27 (1.22–1.31)
Canada	3.28 (1.41–7.21)	3.28 (1.41–7.21)
Norway	2.00 (1.10–3.50)	2.00 (1.10–3.50)
Denmark	1.50 (1.28–1.76)	1.50 (1.28–1.76)
United Kingdom	1.43 (1.11–1.84)	1.43 (1.11–1.84)
Cohort	1.53 (1.28–1.82)	1.38 (1.35–1.41)
Case–control	1.29 (1.00–1.67)	1.37 (1.32–1.43)
Cross-sectional	0.61 (0.43–0.87)	0.61 (0.43–0.87)

## Discussion

### Principal findings

Our findings indicate a positive association between chronic pain (CP) and dementia. Overall, individuals with chronic pain have a 42% increased risk of developing dementia (HR = 1.42, 95% CI 1.23–1.64, *p* < 0.001), suggesting that chronic pain may serve as a risk factor for dementia. Notably, this risk remains consistent regardless of age, indicating that age does not significantly influence the relative risk of developing dementia in individuals with chronic pain. Additionally, we conducted subgroup analyses to explore the effects of different factors on the association between dementia and chronic pain. These factors include subtypes of dementia, subtypes of chronic pain, age, gender, region, follow-up time, educational level, and study design. 

### Comparison with other reviews

A systematic review published in 2020 suggested that chronic pain and specific chronic pain conditions may be associated with an increased risk of developing dementia (Innes and Sambamoorthi 2020). However, most studies included in their review were conducted in Taiwan (China), which may have introduced a geographic bias in the results.

In contrast, our meta-analysis included studies from various countries worldwide, providing a more comprehensive and reliable estimate of the association. We not only conducted a qualitative analysis of the association between different subtypes of chronic pain and dementia, but also performed a quantitative pooling of hazard ratios to draw comprehensive conclusions. This approach minimizes bias and provides a more accurate estimation of the true risk measure. While our study’s overall findings regarding the increased risk of dementia in individuals with chronic pain were consistent with the previous review, we found a 42% increased risk of dementia in chronic pain patients compared to those without chronic pain (HR = 1.42, 95% CI 1.23–1.64, *p* < 0.001). It is important to note that the study samples in our meta-analysis were drawn from diverse cultural, social, and economic backgrounds. However, due to the limited number of studies available from each country or region, we were unable to fully consider the potential impactof these factors. Mendelian randomization analysis could be a valuable approach to further explore the causal relationships in future research.

### Potential interpretations of the results

It is important to interpret subgroup comparisons cautiously due to the small number of studies available. In our subgroup analysis, we found that individuals with chronic pain (CP) were significantly more likely to develop dementia across multiple subtypes of chronic pain, except in one cross-sectional study. In this particular cross-sectional study, dementia was associated with a lower risk of pain. However, due to the nature of cross-sectional study design, it is difficult to determine whether dementia was caused by other factors or if it influenced the perception of pain (Wang et al. 2022).

In this particular cross-sectional study, dementia was associated with a lower risk of pain. However, due to the nature of cross-sectional study design, it is difficult to determine whether dementia was caused by other factors or if it influenced the perception of pain (Wang et al. 2022). Furthermore, a majority of the studies included in our analysis indicated that the risk of dementia increased as the age of individuals with chronic pain advanced (Wang and Liu 2021; Khalid et al. 2022, 2020; Innes and Sambamoorthi 2020; Whitlock et al. 2017; Rouch et al. 2022; Tzeng et al. 2018; Cheng et al. 2022; Kao et al. 2021; Hurh et al. 2022; Morton et al. 2019; Hagen et al. 2014; Islamoska et al. 2020; George et al. 2020; Lee et al. 2019; Kostov et al. 2019; Chen et al. 2018; Huang et al. 2015). This suggests that older age may be a contributing factor to the development of dementia in individuals with chronic pain.

It is important to note that a study conducted by Wang et al. (2022) found that older Americans with dementia are less likely to report the use of pain medications when they experience pain. This finding aligns with previous research highlighting the issue of pain undertreatment among older individuals with dementia (Boltz et al. 2021; Wang et al. 2020; Gilmore-Bykovskyi et al. 2019). Several factors contribute to this phenomenon, including the potential for forgetfulness in reporting pain, lack of recognition of symptoms by clinicians or family members, and the presence of other symptoms such as anxiety, depression, hallucinations, and paranoia, which may lead to medication non-adherence. Additionally, individuals with dementia may face barriers in receiving regular medical checkups, such as mobility issues, forgetfulness, or caregiver constraints. Caregivers themselves may also be unaware of the potential benefits of anti-inflammatory drugs in managing the conditions of these patients. Therefore, healthcare providers need to consider these barriers to appropriate care when treating patients with dementia. While the exact reasons behind this phenomenon cannot be fully determined from research data alone, there is a growing body of literature suggesting that anti-inflammatory drugs may hold promise in preventing Alzheimer’s disease (McGeer and McGeer 1990; Canadian Study of Health and Aging 1994; Breitner et al. 1994). Thus, it is crucial to monitor the occurrence of Alzheimer’s disease and implement preventive measures in individuals with chronic pain to reduce their risk of developing the disease. By remaining vigilant and applying suitable interventions, we may be able to mitigate the impact of chronic pain on cognitive health and improve the overall quality of life for those affected.

Furthermore, Cheng et al. found that depression may be one of the reasons why individuals with trigeminal neuralgia (TN) have a 4.47-fold higher risk of developing dementia compared to those without TN. This finding is consistent with previous research suggesting that the incidence of depression, anxiety, and functional impairment can increase with the severity of pain (Melek et al. 2018). Chronic depression itself is a known factor that predisposes individuals to dementia (Maˇ cianskyt˙e et al. 2011). It has been proposed that the synthesis of NMDA glutamate agonists, such as quinolinic acid and kynurenine metabolites, can lead to oxidative stress and result in a chronic inflammatory state (Leonard 2017). Therefore, it is plausible to suggest that TN may increase the risk of dementia by promoting depression. This novel insight is supported by the study conducted by Cheng et al., which provides additional scientific evidence for this possibility.

The mechanisms underlying the interaction between chronic pain (CP) and dementia are not fully understood. However, current evidence suggests that CP may contribute to an increased risk of dementia through multiple pathways. Several studies have demonstrated that brain pathology associated with dementia can be influenced by pain-related psychopathology. Chronic pain can also provoke dysfunction in the locus coeruleus (LC) and neuroinflammation in dementia patients, leading to neurodegeneration (Monroe et al. 2017, 2012; Cao et al. 2019; Cole et al. 2006, 2011). These changes have been observed in various regions of the brain, including the frontal cortex, where anomalies in the noradrenergic system are frequently observed (Hayashida and Obata 2019). Microglial activation in areas like the LC and increased central neuroinflammation in these regions have also been implicated (Salter and Stevens 2017). The LC plays a crucial role in norepinephrine (NE) synthesis and subsequent neurotransmission in the central nervous system, and dysfunction in the LC-NE signaling pathway has been associated with microglial dysfunction (Gyoneva and Traynelis 2013).

In addition, chronic pain is associated with increased levels of systemic inflammation (Coe et al. 2008; Marchand et al. 2005). Moreover, markers of systemic inflammation can vary among different ethnic and racial groups (Paalani et al. 2011). Inflammation associated with aging has also been linked to dementia and Alzheimer’s disease pathology (Schain and Kreisl 2017; Eldik et al. 2016). Therefore, understanding the underlying mechanisms that contribute to sociodemographic findings requires considering inflammatory factors and their potential impact on the association between chronic pain and dementia.

### Recommendations for research and clinical practice

We found that there is a limited number of studies investigating the association between different subtypes of chronic pain in individuals with dementia, most of which were retrospective cohort studies. To improve pain management in this population, we propose that the first step is to evaluate changes in pain management interventions by monitoring pain intensity. However, distinguishing between behaviors associated with pain and those arising from the dementia process itself can be challenging, posing a significant clinical and research dilemma.

It is essential to recognize that while some pain assessment scales primarily focus on identifying the presence of pain, others, such as the Visual Analog Scale (VAS) and the Numerical Rating Scale (NRS), are specifically designed to evaluate the intensity of pain. Therefore, the choice of a pain assessment scale should align with the specific objectives of the assessment, considering both the identification and quantification of pain. Therefore, we emphasize the use of pain assessment tools that have been validated and tested for responsiveness in well-powered trials of analgesics specifically conducted in moderate to severe dementia patients for future research. Adopting such an approach will allow us to better understand the relationship between different subtypes of chronic pain and dementia, ultimately informing the development of effective pain management strategies in this population. Caregivers in community settings, residential care facilities, nursing homes, and hospitals play a crucial role in enhancing early management and treatment of pain in individuals with dementia and providing appropriate care to prevent adverse outcomes. Finally, further research investigating end-of-life care in this population is necessary to address the unique challenges posed by pain management in individuals with dementia during this stage of life.

### Limitations

Although we conducted a comprehensive literature search and rigorous methodological evaluation, there are some limitations to our findings on the relationship between chronic pain and dementia. Firstly, it’s important to note that there is a wide range of subtypes of chronic pain, and limited original research exists that specifically examines the impact of each subtype on dementia. Therefore, further studies are needed to explore the association between chronic pain subtypes and dementia in more detail. Secondly, pain is subjective and highly personal, making it challenging to measure accurately. Most studies rely solely on self-reported measures of pain, which may be less specific and sensitive compared to clinical trials. This subjectivity can contribute to publication bias and heterogeneity in meta-analyses. However, it’s worth mentioning that heterogeneity is not necessarily invalidating for observational study meta-analyses (Noubiap et al. 2019). Additionally, as dementia progresses slowly and insidiously, it becomes difficult to draw definitive conclusions regarding causality. Furthermore, the studies included in our review were conducted in different countries and regions, where the research sample may be influenced by diverse cultural, social, and economic backgrounds. The limited number of studies from each country/region makes it challenging to assess how these factors may impact the results. In conclusion, while our review provides valuable insights into the relationship between frailty, chronic pain, and dementia, it is important to consider these limitations. Encouraging further research in this area, including studies that examine specific subtypes of chronic pain and consider cultural and social factors, will help obtain a more comprehensive understanding of the association between chronic pain and dementia.

## Conclusions

In conclusion, there is an association between CP and dementia, with individuals with CP being at a higher risk of developing dementia regardless of gender, age, or specific dementia subtypes. Enhancing the quality of life for individuals with CP requires providing education about dementia and implementing regular dementia screening measures. Further extensive research is needed to confirm and extend the findings of this study, identify potential mediating factors, and explore the underlying mechanisms for the observed association between CP and dementia. Through more comprehensive studies, we can gain a better understanding of this relationship and develop targeted interventions to mitigate the impact of CP on dementia.

### Supplementary Information

Below is the link to the electronic supplementary material.

Supplementary file 1 (DOCX 20 KB)

## Data Availability

The original contributions presented in the study are included in the article/Supplementary Material, further inquiries can be directed to the corresponding author/s.
