# Supplementary material for: Association between chronic pain and dementia: a systematic review and meta-analysis
Source: Eur J Ageing. 2024 May 22;21(1):17. doi: 10.1007/s10433-024-00812-2 (PMC11111427; doi:10.1007/s10433-024-00812-2)
Supplement: Supplementary file 1 — (DOCX 20 KB) [file 10433_2024_812_MOESM1_ESM.docx]

| **Table A.** Search strategy for each database: | |
| --- | --- |
| ((("Dementia"[Mesh]) or Dementias or Amentia or Amentias or Senile Paranoid Dementia or Dementias, Senile Paranoid or Paranoid Dementia, Senile or Paranoid Dementias, Senile or Senile Paranoid Dementias or Familial Dementia or Dementia, Familial or Dementias, Familial or Familial Dementias) or (("Alzheimer Disease"[Mesh]) or Alzheimer Dementia or Alzheimer Dementias or Dementia, Alzheimer or Alzheimer's Disease or Dementia, Senile or Senile Dementia or Dementia, Alzheimer Type or Alzheimer Type Dementia or Alzheimer-Type Dementia (ATD) or Alzheimer Type Dementia (ATD) or Dementia, Alzheimer-Type (ATD) or Alzheimer Type Senile Dementia or Primary Senile Degenerative Dementia or Dementia, Primary Senile Degenerative or Alzheimer Sclerosis or Sclerosis, Alzheimer or Alzheimer Syndrome or Alzheimer's Diseases or Alzheimer Diseases or Alzheimers Diseases or Senile Dementia, Alzheimer Type or Acute Confusional Senile Dementia or Senile Dementia, Acute Confusional or Dementia, Presenile or Presenile Dementia or Alzheimer Disease, Late Onset or Late Onset Alzheimer Disease or Alzheimer's Disease, Focal Onset or Focal Onset Alzheimer's Disease or Familial Alzheimer Disease (FAD) or Alzheimer Disease, Familial (FAD) or Familial Alzheimer Diseases (FAD) or Alzheimer Disease, Early Onset or Early Onset Alzheimer Disease or Presenile Alzheimer Dementia))AND((("Chronic pain" [Mesh]) or Chronic Pains or Pains, Chronic or Pain, Chronic or Widespread Chronic Pain or Chronic Pain, Widespread or Chronic Pains, Widespread or Pain, Widespread Chronic or Pains, Widespread Chronic or Widespread Chronic Pains)OR(("Pain, Intractable" [Mesh]) or Intractable Pain or Intractable Pains or Pains, Intractable or Refractory Pain or Pain, Refractory or Pains, Refractory or Refractory Pains)) | PUBMED |
| **#15**  #7 AND #14  [1,230](https://www.embase.com/)  **#14**  #10 OR #13  [417,244](https://www.embase.com/)  **#13**  #11 OR #12  [230,987](https://www.embase.com/)  **#12**  **'alzeimer disease'**:ab,ti OR **'alzeimers disease'**:ab,ti OR **'alzheimer dementia'**:ab,ti OR **'alzheimer fibrillary change'**:ab,ti OR **'alzheimer fibrillary lesion'**:ab,ti OR **'alzheimer neurofibrillary change'**:ab,ti OR **'alzheimer neurofibrillary degeneration'**:ab,ti OR **'alzheimer neuron degeneration'**:ab,ti OR **'alzheimer perusini disease'**:ab,ti OR **'alzheimer sclerosis'**:ab,ti OR **'alzheimer syndrome'**:ab,ti OR **'alzheimers disease'**:ab,ti OR **'cortical sclerosis, diffuse'**:ab,ti OR **'dementia, alzheimer'**:ab,ti OR **'diffuse cortical sclerosis'**:ab,ti OR **'late onset alzheimer disease'**  [4,418](https://www.embase.com/)  **#11**  **'alzheimer disease'**/exp  [230,309](https://www.embase.com/)  **#10**  #8 OR #9  [416,811](https://www.embase.com/)  **#9**  **'amentia'**:ab,ti OR **'demention'**  [104](https://www.embase.com/)  **#8**  **'dementia'**/exp  [416,752](https://www.embase.com/)  **#7**  #3 OR #6  [108,704](https://www.embase.com/)  **#6**  #4 OR #5  [13,927](https://www.embase.com/)  **#5**  **'pain, intractable'**:ab,ti  [73](https://www.embase.com/)  **#4**  **intractable** AND **pain**  [13,927](https://www.embase.com/)  **#3**  #1 OR #2  [97,114](https://www.embase.com/)  **#2**  **'chronic intractable pain'**:ab,ti OR **'pain, chronic'**  [2,003](https://www.embase.com/)  **#1**  **'chronic pain'** | EMBASE |
| (((Dementia or Dementias or Amentia or Amentias or Senile Paranoid Dementia or Dementias, Senile Paranoid or Paranoid Dementia, Senile or Paranoid Dementias, Senile or Senile Paranoid Dementias or Familial Dementia or Dementia, Familial or Dementias, Familial or Familial Dementias) or (Alzheimer Disease or Alzheimer Dementia or Alzheimer Dementias or Dementia, Alzheimer or Alzheimer's Disease or Dementia, Senile or Senile Dementia or Dementia, Alzheimer Type or Alzheimer Type Dementia or Alzheimer-Type Dementia (ATD) or Alzheimer Type Dementia (ATD) or Dementia, Alzheimer-Type (ATD) or Alzheimer Type Senile Dementia or Primary Senile Degenerative Dementia or Dementia, Primary Senile Degenerative or Alzheimer Sclerosis or Sclerosis, Alzheimer or Alzheimer Syndrome or Alzheimer's Diseases or Alzheimer Diseases or Alzheimers Diseases or Senile Dementia, Alzheimer Type or Acute Confusional Senile Dementia or Senile Dementia, Acute Confusional or Dementia, Presenile or Presenile Dementia or Alzheimer Disease, Late Onset or Late Onset Alzheimer Disease or Alzheimer's Disease, Focal Onset or Focal Onset Alzheimer's Disease or Familial Alzheimer Disease (FAD) or Alzheimer Disease, Familial (FAD) or Familial Alzheimer Diseases (FAD) or Alzheimer Disease, Early Onset or Early Onset Alzheimer Disease or Presenile Alzheimer Dementia))AND((Chronic pain or Chronic Pains or Pains, Chronic or Pain, Chronic or Widespread Chronic Pain or Chronic Pain, Widespread or Chronic Pains, Widespread or Pain, Widespread Chronic or Pains, Widespread Chronic or Widespread Chronic Pains or Pain, Intractable or Intractable Pain or Intractable Pains or Pains, Intractable or Refractory Pain or Pain, Refractory or Pains, Refractory or Refractory Pains))) | **Web of science** |

|  |
| --- |
